# VBM Reveals Brain Volume Differences between Parkinson’s Disease and Essential Tremor Patients

**DOI:** 10.3389/fnhum.2013.00247

**Published:** 2013-06-14

**Authors:** Ching-Hung Lin, Chun-Ming Chen, Ming-Kuei Lu, Chon-Haw Tsai, Jin-Chern Chiou, Jan-Ray Liao, Jeng-Ren Duann

**Affiliations:** ^1^Biomedical Engineering R&D Center, China Medical University, Taichung, Taiwan; ^2^Biomedical Electronics Translational Research Center, National Chiao Tung University, Hsinchu, Taiwan; ^3^Department of Psychology, Soochow University, Taipei, Taiwan; ^4^Department of Electrical Engineering, National Chung Hsing University, Taichung, Taiwan; ^5^Department of Neurology, China Medical University Hospital, Taichung, Taiwan; ^6^School of Medicine, Medical College, China Medical University, Taichung, Taiwan; ^7^School of Medicine, Graduate Institute of Clinical and Medical Science, China Medical University, Taichung, Taiwan; ^8^Institute for Neural Computation, University of California San Diego, La Jolla, CA, USA

**Keywords:** voxel-based morphometry, Parkinson’s disease, essential tremor, DARTEL VBM, basal ganglia, cerebellum, ventro-posterior-lateral thalamus, middle temporal gyrus

## Abstract

Symptoms of essential tremor (ET) are similar to those of Parkinson’s disease (PD) during their initial stages. Presently, there are few stable biomarkers available on a neuroanatomical level for distinguishing between these two diseases. However, few investigations have directly compared the changes in brain volume and assessed the compensatory effects of a change in the parts of the brain associated with PD and with ET. To determine the compensatory and/or degenerative anatomical changes in the brains of PD and ET patients, the present study tested, via two voxel-based morphometry (VBM) approaches (Basic vs. DARTEL VBM processing), the anatomical brain images of 10 PD and 10 ET patients, as well as of 13 age-matched normal controls, obtained through a 3T magnetic resonance scanner. These findings indicate that PD and ET caused specific patterns of brain volume alterations in the brains examined. In addition, our observations also revealed compensatory effects, or self-reorganization, occurring in the thalamus and the middle temporal gyrus in the PD and ET patients, due perhaps in part to the enhanced thalamocortical sensorimotor interaction and the head-eye position readjustment, respectively, in these PD and ET patients. Such a distinction may lend itself to use as a biomarker for differentiating between these two diseases.

## Introduction

Patients with Parkinson’s disease (PD) and essential tremor (ET) share some common symptoms (such as resting tremor) during their initial stages of illness. However, these two diseases involve different internal mechanisms and therefore require different clinical treatments (Draganski and Bhatia, 2010; Watts et al., 2011). Numerous studies have utilized brain imaging to probe alterations in the structural and functional organization of patients with PD (Kassubek et al., 2002; Brenneis et al., 2003; Burton et al., 2004; Price et al., 2004; Chebrolu et al., 2006; Beyer et al., 2007; Ramirez-Ruiz et al., 2007; Bouchard et al., 2008; Feldmann et al., 2008; Ibarretxe-Bilbao et al., 2008, 2009a, 2011a,b; Benninger et al., 2009; Camicioli et al., 2009; Cardoso et al., 2009; Jubault et al., 2009; Martin et al., 2009; Wattendorf et al., 2009; Agosta et al., 2010a,b; Bruggemann et al., 2010; Hamasaki et al., 2010; Focke et al., 2011). Over the past decade, the voxel-based morphometry (VBM) technique has been applied often for studying brain volume changes in PD and other degenerative brain diseases. These studies have shown brain atrophy to exist in many cortical and subcortical regions, particularly in the basal ganglia [BG, which contains five nuclei: the caudate nucleus (CN), the putamen (PT), the globus pallidus (GP), the sub-thalamic nucleus (STN), and the substantia nigra (SN)] within the PD group (Gazzaniga et al., 2008; Mink, 2008; Dum and Strick, 2009; Watts et al., 2011). However, these PD-VBM studies have not yet drawn any congruent conclusions (see Table S1 in Supplementary Material). It is worth noting that five PD-VBM studies have pinpointed not only PD-related brain volume loss in certain brain regions but also volume increase in certain other areas (Kassubek et al., 2002; Reetz et al., 2009, 2010; Cerasa et al., 2011; Jubault et al., 2011). Among the brain areas that increased in volume are the frontal lobe, the temporo-parietal junction (TPJ), the parietal lobe, the insula (IN), the anterior cingulate cortex (ACC), the BG, and the thalamus, as has been reported in the literature. Nevertheless, few of these findings were wholly consistent with each other (see Table 1). Therefore, this issue of brain volume increases detected with VBM in PD groups will require further examination.

**Table 1:** **Summary of brain volume increased with VBM in Parkinson’s disease studies**.

Changed Brain Area	Frontal lobe	Parieto-temporo-occipital association cortex	Parietal lobe	Insula	Anterior cingulate cortex	Basal ganglia	Thalamus
Research Group
Kassubek et al. (2002)							Ventralis intermedius (VIM)
Reetz et al. (2009)						Right globus pallidus externus (sPARKINMC) and right putamen (iPD)	
Reetz et al. (2010)		Parieto-temporo-occipital association cortex (asymptomatic Parkin and ATP13A2 MC)				Striatum (asymptomatic Parkin, PINK1, ATP13A2)	
Cerasa et al. (2011)	Bilateral inferior frontal gyrus (in dyskinetic patients)						
Jubault et al. (2011)	Right superior frontal gyrus (surface area)		Bilateral parietal lobule (surface area)	Left insular cortex (surface area)	Left cingulate cortex (surface area)		

On the other hand, relatively few investigations using a VBM technique have been conducted in order to identify volumetric changes in brain structure associated with ET (Daniels et al., 2006; Quattrone et al., 2008; Benito-Leon et al., 2009; Cerasa et al., 2009; Bagepally et al., 2010). Moreover, the ET-VBM studies that have been conducted have not yet drawn any congruent conclusions (see Table 2).

**Table 2:** **Summary of brain volume change observed with VBM in Essential Tremor Studies**.

Changed Brain Area	Frontal lobe	Temporal lobe	Parietal lobe	Limbic lobe	Occipital lobe	Insula	Basal ganglia	Thalamus	Hippocampus	Pons and medulla	Cerebellum
Research Group
Daniels et al. (2006)		X	X		X Right middle						No consistent result
Quattrone et al. (2008)											X Vermis
Benito-Leon et al. (2009)	X Right		X Bilateral			X Right					X Bilateral
Cerasa et al. (2009)											X
Bagepally et al. (2010)	X Bilateral	X Left middle	X Right superior		X Bilateral		X	X			X

In fact, the known movement disorders of PD and ET patients have largely been linked to the damage done to motor loops within the central nervous system (Draganski and Bhatia, 2010; Watts et al., 2011). Traditional motor-system studies have identified two motor programing circuits that are important for skill learning and for action modulation, namely, the basal ganglia-thalamocortical (BTC) loop (Gazzaniga et al., 2008; Mink, 2008; Dum and Strick, 2009; Watts et al., 2011) and the cerebello-thalamocortical (CTC) loop (Gazzaniga et al., 2008; Mauk and Thach, 2008; Dum and Strick, 2009; Watts et al., 2011).

The BTC loop is linked mainly to the BG, the supplementary motor area (SMA) (Ash et al., 2011), the thalamus, the prefrontal cortex, and partially to the temporal cortex, the hippocampus, and a few occipital regions. This loop is activated largely by the internal motivation, and is also involved in proficient motor behavior. On the other hand, the CTC pathway contains several cortico-subcortical regions, including the cerebellum, premotor cortex, thalamus, dorsolateral prefrontal cortex (DLPFC), and parietal cortex. The actions from the CTC loop regulate the movement system in the initial period of motor learning. This circuitry plays a critical role in conducting the actions guided by environmental cues (i.e., external stimuli) (Gazzaniga et al., 2008; Mink, 2008; Dum and Strick, 2009).

Previously, gray-matter volume changes in the BG and SN had been reported to be significantly associated with PD (Mink, 2008; Dum and Strick, 2009). Nevertheless, the manner in which the volumetric changes in the brain areas of the BTC loop relate to PD is still largely incoherent (see Table S1 in Supplementary Material). Although previous studies have revealed gray-matter volumetric changes in the cerebellum and the thalamus (Quattrone et al., 2008; Benito-Leon et al., 2009; Bagepally et al., 2010) in the ET patient’s brain, the manner in which volumetric changes in the brain areas along the CTC loop are associated with ET is still largely unknown (see Table 2).

As certain motor circuitries in the brains of PD and ET patients gradually degenerate, different motor control loops are likely to be utilized. Consequently, certain brain areas in these alternative pathways might then become more active, so as to compensate for the loss of function, and may thus increase in size. The present study, therefore, examines the neuroanatomical differences between the PD patients, the ET patients, and the age-matched normal control subjects using two VBM methods: the basic VBM and DARTEL (Diffeomorphic Anatomical Registration Through Exponentiated Lie Algebra) VBM (Ashburner and Friston, 2000; Ashburner, 2010) in SPM.

This study aims to compare the morphological changes of the brains between the three subject groups [namely, the PD, the ET, and the CT (normal control subjects)] and to confirm the morphometry results using two different VBM methods so as to diminish possible false positive findings (Chumbley and Friston, 2009). Here we hypothesized that the conjunctive regions revealed by the two methods should be relatively stricter than that of any single method. Actually, the DARTEL VBM is based on the basic VBM but more precise in the preprocess procedure than in the basic VBM: first, the processing of DARTEL VBM uses an affine regularization with the East Asian brain template, which fits better the patient/subject populations in this study. Furthermore, during the spatial normalization step, the DARTEL VBM creates brain templates according to the anatomical images of specific subject groups, and then normalizes these templates with respect to the standard Montreal Neurological Institute (MNI) template (305 T1) (Talairach and Tournoux, 1988). This additional procedure of the DARTEL VBM makes its spatial normalization more precise than that of the basic VBM (Ashburner and Friston, 2000; Ashburner, 2010). In line with this, we have found more brain areas with significant volumetric morphometric differences using the DARTEL VBM method in this study.

More specifically, this study would like to test for any specific patterns of compensatory volumetric changes, more specifically of volume increase, as opposed to the atrophic effects indicated by the volume loss in the brain areas along the BTC and CTC pathways, each associated with different patient populations.

## Materials and Methods

### Subjects

The study recruited 33 subjects – 10 ET patients (five male and five female, mean age = 63.4 years; SD = 8.71 years), 10 PD patients (eight male and two female, mean age = 67.30; SD = 8.77), and 13 age-matched normal controls (nine male and four female, mean age = 65.31 years; SD = 11.09 years). The PD and ET patients were screened with standard clinical tests (PD: Unified Parkinson’s Disease Rating Scale (UPDRS); ET: Intention Tremor Score) by neurologists. The relative demographic characteristics of each group are listed in Table 3. Each subject gave informed consent before the MRI examination, the Institutional Ethics Committee of China Medical University Hospital approved the study (IRB No. DMR98-IRB-290), and the experiment was conducted according to the Declaration of Helsinki.

**Table 3:** **Demographic characteristics and clinical assessment scores in PD, ET, and healthy controls**.

Group	PD	ET	Control
Number of subjects	10	10	13
Age (year)	67.30 ± 8.77	63.40 ± 8.71	65.31 ± 11.09
Gender (male/female)	8M/2F	5M/5F	9M/4F
Disease duration (year)	2.85 ± 2.47	15.20 ± 7.91	–
UPDRS-I	2.90 ± 1.97	–	–
UPDRS-II	8.00 ± 3.83	–	–
UPDRS-III	22.50 ± 8.29	–	–
H&Y stage	2.20 ± 0.26	–	–
intention tremor score	–	1.40 ± 0.52	–

### Acquisition of MRI data

The MRI examinations were conducted using a quadrature head coil on a Signa HDx 3.0T MRI scanner (GE, Milwaukee, WI, USA). During each MRI scan, a foam-rubber cushion was used for fixing the head of the subject in place, so as to minimize any voluntary head movement. In order to capture detailed anatomical information, a high-resolution T1-weighted image was utilized, using a 3-D gradient-echo pulse sequence, and a Modified Driven Equilibrium Fourier Transform (MDEFT) with the following imaging parameters: image matrix = 256 × 256; FOV = 224 mm × 224 mm; number of slices = 170; slice thickness = 1 mm; flip angle = 12° and TR/TE/TI (inversion time) = 7.372 ms/2.74 ms/650 ms.

### Data processing and analysis

**Figure 1:**
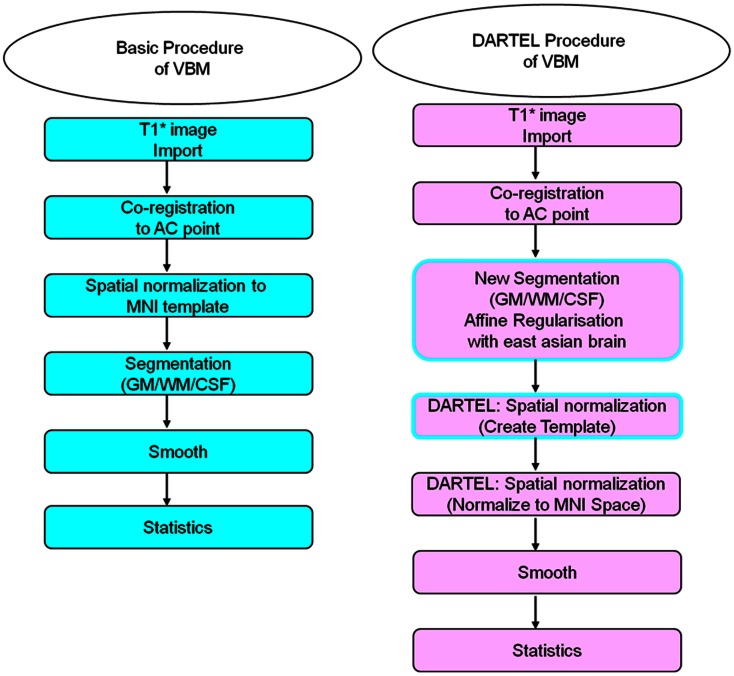
**The Procedures of image processing with basic and DARTEL VBM methods**. Here we defined the preprocessing of basic VBM method that first, coregistering all subjects’ anterior commercial (AC) point to the central point of space and launched the spatial normalization to MNI template before the segmentation for gray matter, white matter, and cerebral spinal fluid. Finally, the 8 mm full-width-half-maximum (FWHM) was enrolled here for smoothing. On the other hand, the first two steps of preprocessing (T1* image import and correct AC point) of DARTEL VBM is as same as that of basic VBM. However, the DARTEL VBM method launched a new segmentation processing first, in an affine regularization with East Asian brain. Furthermore, the DARTEL VBM method also provided a function to create a template by integrating the sampled subjects’ brain for spatial normalization and finally normalized to the MNI template for position localization. The other image-processing step and statistical testing were similar to the basic VBM method.

Statistical Parametric Mapping (SPM8) (Department of Cognitive Neurology, London, UK) performed with (MATLAB 2010a, 2010) (MathWorks, Boston, MA, USA), was utilized for preprocessing and analyzing the MRI data. The statistical results were then presented using SPM8 and xjView 8.0 (Human Neuroimaging Lab, Baylor College of Medicine, Houston, TX, USA). Both basic and DARTEL VBM image-processing techniques, as described in the literature (Ashburner and Friston, 2000; Good et al., 2001; Ridgway et al., 2008; Ashburner, 2010; Kurth et al., 2010), were adopted for evaluating differences in volumetric changes of various brain areas for the three subject groups. Figure 1 illustrates the flowchart for the two image-processing procedures.

Briefly, the anatomical MRI images were co-registered, normalized, and spatially smoothed using an 8-mm full-width-at-half-maximum (FWHM) Gaussian kernel in SPM8. The random effect model was adopted for the between-group analysis in order to compute any differences between the two population groups. Brain areas with significant volumetric differences were identified using the statistical threshold of uncorrected *P* = 0.05, with spatial extent *K* ≥ 30 voxels. Talairach and Tournoux’s (1988) 3-D brain stereotaxic system, with a MNI template (305 T1) in SPM8, and xjView 8 were used to determine the coordinates of local *T*-maxima. The brain areas with significant differences between population groups were reported only when they were identified by both VBM methods.

## Results

### Changes in volume of brain areas for PD patients as compared to normal subjects (PD vs. CT)

**Figure 2:**
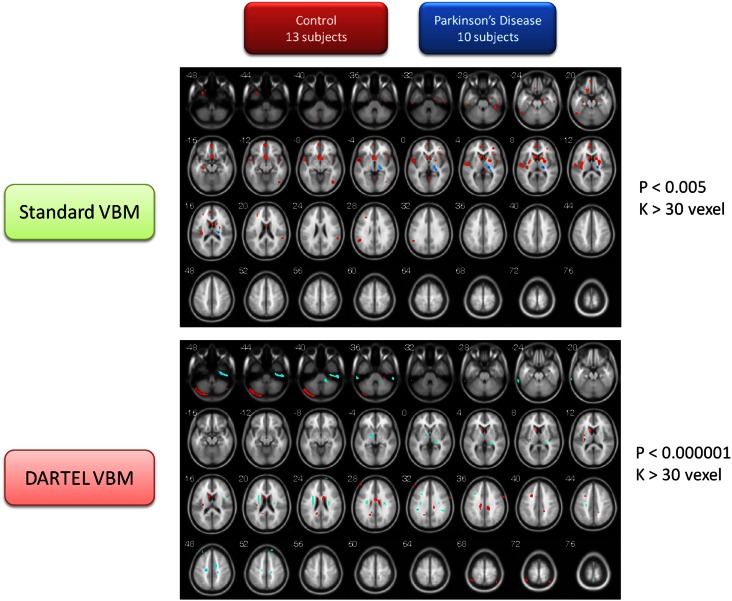
**Contrast maps of volume changes between healthy controls and Parkinson’s disease with two VBM methods**. The warm color represents the gray-matter volume of healthy controls larger than that of PD. Conversely, the cold color marks the gray-matter volume of PD larger than that of healthy controls. The basic VBM method revealed that PD patients have large atrophy in basal forebrain, IN, frontal cortex, temporal cortex, and several occipital regions. The DARTEL VBM method with a critically significant-level also demonstrated the basal ganglia atrophy in PD patients, but the other atrophy regions are not totally consistent with the observation using basic VBM method. Conversely, the two methods consistently pinpointed that the posterior part of thalamus in PD patients is larger than that of normal controls.

Both VBM methods consistently found brain volumes to have decreased in the brain areas of the lentiform nucleus (LN), the IN, the middle frontal gyrus (MFG), and the cerebellar vermis in the PD group when compared to the normal controls. The details of brain areas with significant volumetric differences are listed in Table S2 in Supplementary Material. Table S2A in Supplementary Material shows the brain areas in which volume sizes are smaller in PD patients than in normal controls. The upper part of the table shows the results derived using the basic VBM, and the lower part shows the results using the DARTEL VBM method. The shaded areas in the table highlight the common results obtained from both VBM methods. Similarly, the brain areas with increased volumes in PD patients, as compared to the normal controls, are listed in Table S2B in Supplementary Material. Both VBM methods consistently identified the gray matter of the PD patients’ thalamus (ventral posterior lateral nucleus, VPL) as being larger than that of the normal control. The contrasting image between these two subject populations is depicted in Figure 2.

### Changes in volume of brain areas for ET patients as compared to normal subjects (ET vs. CT)

**Figure 3:**
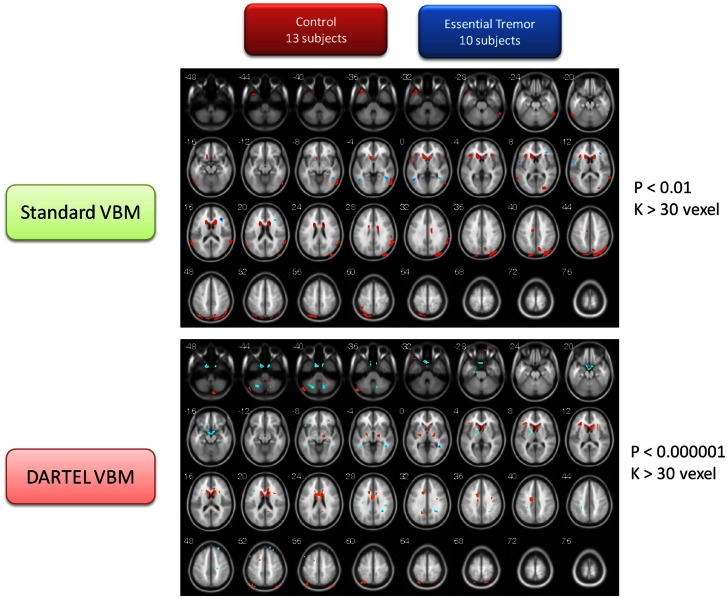
**Contrast maps of volume changes between healthy controls and essential tremors with two VBM methods**. The warm color represents the gray-matter volume of healthy controls larger than that of ET. On the other hand, the cold color marks the gray-matter volume of ET larger than that of healthy controls. The basic VBM method revealed that ET patients have large atrophy in basal ganglia, particularly the caudate nucleus. Furthermore, IN, temporal cortex, and parietal cortex were also observed. On the other hand, the DARTEL VBM method also confirmed that the caudate nucleus serious atrophy in ET patients. Additionally, the parietal cortex, cerebellum, and thalamus atrophy in ET patients was observed in DARTEL VBM method. Conversely, the two methods congruently identified that the bilateral MTG was significantly large than that of normal controls.

This portion of the study draws attention to the volumetric changes in the brains of ET patients compared to age-matched normal subjects. These two VBM methods consistently found that the brain volumes of the ET group were significantly smaller in many brain regions than that of control group, including the caudate body, the middle temporal pole (L), the IN, the precuneus (L), and the superior temporal gyrus (STG). The detailed brain atrophy for the ET group is listed in Table S3A in Supplementary Material (the same color row marked similar brain regions for both methods). These two VBM methods, however, persistently identified the gray matter of the middle temporal gyrus (MTG), and the precentral gyrus in the ET group, to be larger than that of the normal control group (for detailed volume differences, please refer to Table S3B in Supplementary Material). The same color row marked similar brain regions for both methods. The contrast maps between the two groups are shown in Figure 3.

### Changes in volume of brain areas for PD patients as compared to ET patients (PD vs. ET)

**Figure 4:**
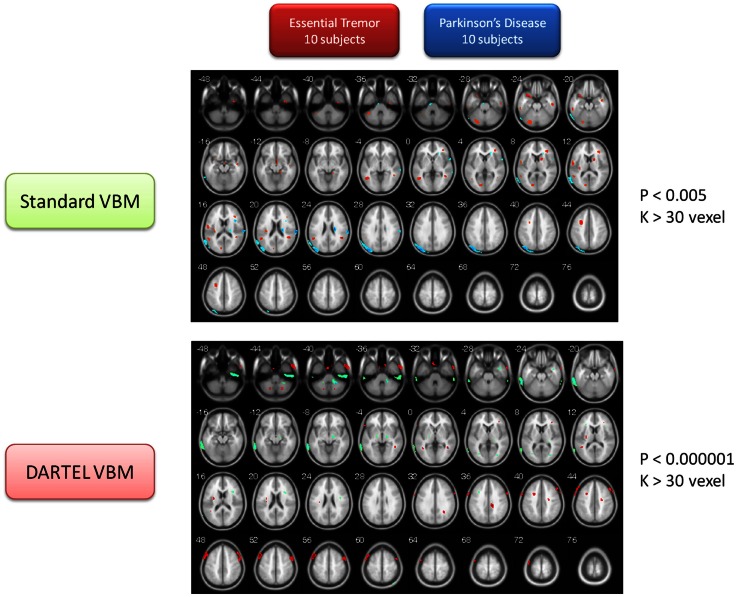
**Contrast maps of volume changes between essential tremors and Parkinson’s disease with two VBM methods**. In this figure, the warm color represents the gray-matter volume of ET larger than that of PD, alternatively, the cold color marks the gray-matter volume of PD larger than that of ET. The additional comparison between the PD and ET patients indicated that the MTG, MFG, IN, temporal lobe, and cerebellum of ET were larger than that of PD in both VBM methods (see also Figure 3). Moreover, the both methods demonstrated that the parietal cortex, temporal cortex, and thalamus of PD were larger than that of ET. The finding on thalamus is consistent with the previous result in comparison between PD patients and healthy control (see also Figure 2).

In addition, this study also provided the volume of gray matter so as to compare between the two types of motor disorder (PD vs. ET). The utilization of the two VBM methods mentioned above served to confirm that the brain volume of the ET group was significantly smaller than that of the PD group in many brain regions, including the thalamus (VPL) and the MTG (R). In addition, both VBM methods persistently identified the gray matter of the MFG, the MTG, the cerebellum posterior lobe (Crus 1), and the IN in the ET group, to be larger than that of the PD group (for details about the volume change between the ET and PD groups, please refer to Tables S4A,B in Supplementary Material) (The identical color row represented consistent findings between both methods). The contrast maps for the two groups are depicted in Figure 4.

## Discussion

In this study we observed how the atrophic regions in PD groups are not only located in BTC loops but in CTC loops as well. The volume alterations associated with ET, on the other hand, are located in both BTC and CTC circuitries. As a result, differentiating between these two neurodegenerative movement disorders based solely on brain morphological alterations might not be the most effective solution. However, such an overlap might be the reason as to why these two movement disorders share common symptoms of resting tremor, at least during the early stages of their onset. It is also worth noting that the volume increase of thalamus in PD groups in the present study is consistent with the hypothesized compensatory models of BTC. Conversely, the volume increase of the MTG in ET groups varies from what was predicted in the hypothesized compensatory model of the CTC. The function of the MTG was regarded as being mainly the processing of motion perception and of ocular movement. Nevertheless, some ET patients have head tremor over the long term and may need to compensate by keeping their eye field stabilized. The MTG of ET patients might therefore need to be constantly activated so as to integrate their body-environment sensation and coordinate their head- and eye-centric systems. As a result, the MTG may very likely enlarge in size in response to such long-term compensatory effects.

This study utilized two VBM methods for identifying the atrophic and compensatory effects in PD and ET patients, as compared to healthy controls. Both VBM methods consistently found that the PD patients have gray-matter atrophy in the BG, IN, MFG, and the cerebellum. Additionally, both methods also congruently identified the posterior part of thalamus as having increased in volume when compared to that of normal controls. We also observed that the gray matter of the caudate body, middle temporal pole, IN, precuneus, and the STG decreased, but that of the MTG increased in ET patients. The compensatory effect on PD and ET patients, during both VBM methods, was discussed less in previous brain volumetric morphology studies. Here we provide an alternative viewpoint, particularly as regards the compensatory effect on certain brain regions (e.g., the thalamus in the PD group; the MTG in the ET group) that may help to reconsider the mechanism of movement symptoms during variant stages.

### PD vs. CT

This study’s observations on brain atrophy, including that in the LN, the IN, the MFG, and the cerebellum (vermis) in the PD patients group, using both VBM methods, is consistent with a number of previous functional and structural MRI studies (Higginson et al., 2001; Brenneis et al., 2003; Nakajima et al., 2003; Price et al., 2004; Grafton et al., 2006; Pahwa et al., 2006; Phillips et al., 2006; Feldmann et al., 2008; Ibarretxe-Bilbao et al., 2008, 2009a,b,c; Borghammer et al., 2009; Camicioli et al., 2009; Cardoso et al., 2009; Pereira et al., 2009, 2011; Wattendorf et al., 2009; Hamasaki et al., 2010) (see Figure 2; Table S1 in Supplementary Material). It is worth noting that the gray matter of the thalamus (VPL) of the PD group is larger than that of the control group, as shown by both VBM methods. The present finding on thalamus enlargement is not noted in previous PD-related studies, except for that of Kassubek et al. (2002). This finding is not inaccessible, as conventional literature has consistently demonstrated how the thalamus receives inhibitory inputs from the GPi (Internal Part of Globus Pallidus) and the SN, and then projects excitatory output to the cortex (Lubar and Bahler, 1976). In line with the BTC loop, the inhibitory signal from the BG (GPi and SN) is weaker in PD patients as compared to healthy controls. Over the long run, therefore, thalamus enlargement may occur due to a decreased inhibitory signal (Deogonkar et al., 2011). Consequently, the excitatory output from the thalamus to the cortex may become too large and the signal may scatter in an unstable manner. Conversely, this phenomenon of thalamus enlargement may contribute to the aftereffects of medical treatment. Most patients were given l-DOPA treatment for many years to compensate for the function loss of BTC loops. Therefore, a number of stereotaxic surgeries and deep brain stimulation techniques on the thalamus can improve the symptoms of PD patients (Deogonkar et al., 2011).

### ET vs. CT

Atrophy in the CN, middle temporal pole, IN, Precuneus, and the STG appeared consistently when using both methods (Table S3A in Supplementary Material). The present observation indicates that the resting tremor of ET patients might be due to gradual damage to the BTC loop. On the other hand, the present analysis also demonstrates that the MTG and the precentral gyrus have increased the volume of gray matter within the ET group (Table S3B in Supplementary Material). This MTG was known to be in response to motion detection and eye-movement control. The volume increase of MTG might be generated by the visual-motor coordination function in ET patients with head tremor.

### PD vs. ET

An additional analysis was launched at this point for testing volume differences between PD and ET patients. The findings were mostly consistent with the two previous comparisons to the control group (PD vs. CT; ET vs. CT). The PD group has a relatively larger volume growth of the VPL thalamus and the MTG (Figure 4; Table S4A in Supplementary Material). The ET group, on the other hand, possessed a relatively larger volume increase in gray matter in the MFG, the MTG, the cerebellum posterior lobe (Crus 1), and the IN (Figure 4; Table S4B in Supplementary Material). The increased volume of these motor control regions may imply that these brain regions are involved in compensating for the effect of motor function over the long-term. For example, several patients with head tremor type suffered from uncomfortable involuntary head-motions, which meant the eye-movement control system had to expend more effort on immediate correction in response to the external world. Nevertheless, medical treatment may be an important confounding factor in interpreting the compensation effects of brain volume. This confounding factor will require a relatively larger amount of data for further clarification in the near future.

This study, by using two analysis methods, was able to confirm that gray-matter atrophy (in the BG), and its compensatory effects (in the posterior part of the thalamus) in PD patients, conform to what was predicted by the BTC hypothesis. However, the ET patients’ gray-matter atrophic effect related mostly to the BG and the neocortex, while its compensatory effects were observed mainly in the MTG, which is not completely congruent with the CTC hypothesis. Additionally, the brain volume change of ET patients was due mostly to the large atrophy of the CN. This may imply that the ET and PD partially share the damage mechanism in BTC loops, which, in turn, might explain, at least in part, their similar external symptoms (e.g., resting tremor).

The present study directly compared brain morphology between the three subject groups: the PD, the ET, and the normal control subjects. This study, however, was limited by the sample size of each group. To prevent an over-interpretation of its results, we employed the two VBM methods to cross validate the findings. Although the two VBM methods had slightly different analysis procedures, they resulted in fairly similar brain regions showing significant morphological changes associated with these two diseases. Both methods found several overlapping brain regions with disease-related volumetric differences from patients in the same groups. The DARTEL VBM, however, used a more precise spatial normalization procedure and thus found a relatively larger number of brain areas than the basic VBM. Therefore, we used different statistical criteria (uncorrected *p* < 0.005 for the standard VBM and uncorrected *p* < 0.000001 for the DARTEL VBM) for screening significant voxels in both methods. It is also worth noting that even with limited sample size for each of the patient/subject populations, the DARTEL VBM method could still find quite a few significant brain regions with significant morphometric differences among patient groups under a stringent statistical criterion of uncorrected *p* < 0.000001. Using conjunction of these two VBM methods should be able to further address the problem of false positive caused by multiple comparisons in this study.

Over the past decade, the VBM methods and the standard of procedure of performing VBM analysis are still under continual revision and validation (see Table S1 in Supplementary Material; Tables 1 and 2). This is especially true when applying VBM to the patient populations with neurodegenerative diseases, such as PD and ET. Different degrees of atrophy in these patient populations might cause problems in spatial normalization at the boundary of the brain due at least in part to the intrusive degenerative portions. How such degenerative brains in the patient populations may affect the result of VBM analysis remains to be further explored in detail.

## Conclusion

As shown in both VBM analyses, the results reveal both decreased and increased volumes in brain areas that are involved in both BTC and CTC circuitries. Decreases in the volumes of the BG, the IN, and in many cortical regions, are consistent with previous PD studies. The present study also found that the volume increase in the PD group occurs in the VPL thalamus. Serious atrophy in the LN, the IN, the temporal, and the parietal cortex, on the other hand, is observed in the ET group. Notably, the increase in volume of the MTG was very evident in the ET group. The present observation is consistent with a number of PD- and ET-VBM studies, which state that these long-term movement disorders are associated not only with the atrophy of brain regions involved in motor control loops, but also with the enlargement of certain other brain regions. Such an enlargement may reflect a compensatory effect in response to the damaged motor control loops, the BTC, or to the CTC loops. As a result, the brain regions that increase in volume may play a more specific role following any brain damage caused by PD and ET, and may therefore be used as biomarkers for the diagnosis of these movement disorders, as well as for their clinical assessment.

## Authors Contribution

Ching-Hung Lin, Chun-Ming Chen, and Jeng-Ren Duann contributed to the conceptual innovation, literature review, data interpretation, and drafting of the preliminary manuscript. Chun-Ming Chen, Jeng-Ren Duann acquired all image data. Ching-Hung Lin provided the image processing as well as statistical analysis. Ming-Kuei Lu and Chon-Haw Tsai were responsible for patients screening and provided some critical observation and discussion. Chon-Haw Tsai and Jin-Chern Chiou set up all experimental conditions for this study. Jeng-Ren Duann arranged all imaging experiments and finalized the MRI data interpretations with Ching-Hung Lin. Jan-Ray Liao provided some concepts of digital image processing in MR image reconstruction and data analysis. All authors have consented to the submission and publication of the manuscript.

## Conflict of Interest Statement

The authors declare that the research was conducted in the absence of any commercial or financial relationships that could be construed as a potential conflict of interest.

## Supplementary Material

The Supplementary Material for this article can be found online at http://www.frontiersin.org/Human_Neuroscience/10.3389/fnhum.2013.00247/abstract

## Abbreviations

ACC, anterior cingulate cortex; BG, basal ganglia; BTC, basal ganglia-thalamocortical circuit; CN, caudate nucleus; CTC, cerebello-thalamocortical; DLPFC, dorsolateral prefrontal cortex; ET, essential tremor; GP, globus pallidus; IN, insula; LN, lentiform nucleus; MFG, middle frontal gyrus; MTG, middle temporal gyrus; PD, Parkinson’s disease; PT, putamen; SN, substantia nigra; STG, superior temporal gyrus; STN, sub-thalamic nucleus; TPJ, temporo-parietal junction; VBM, voxel-based morphometry; VPL, ventral posterior lateral nucleus.
