# Supplementary material for: VBM Reveals Brain Volume Differences between Parkinson’s Disease and Essential Tremor Patients
Source: Front Hum Neurosci. 2013 Jun 14;7:247. doi: 10.3389/fnhum.2013.00247 (PMC3682128; doi:10.3389/fnhum.2013.00247)
Supplement: Supplementary file 1 [file 46476_Duann_DataSheet1.DOC]

**Supplementary Table 1**  **Summary of brain volume change observed with VBM in Parkinson’s Disease Studies.**

| **Changed Brain**  **Area**  **Research Group** | **Frontal Lobe** | **Temporal**  **Lobe** | **Parietal Lobe** | **Limbic**  **Lobe** | **Occipital**  **Lobe** | **Insula** | **Anterior Cingulate**  **Cortex** | **Basal**  **Ganglia** | **Thalamus** | **Hippocampus** | **Amygdala** | **Pons and Medulla** | **Cerebellum** |
| --- | --- | --- | --- | --- | --- | --- | --- | --- | --- | --- | --- | --- | --- |
| Kassubek  (2002) Neuroscience Letters |  |  |  |  |  |  |  | ╳  (nucleus ventralis intermedius of the thalamus) |  |  |  |  |  |
| Brenneis et al. (2003) Movement Disorder | ╳  (primary sensorimotor cortices, supplementary motor, right premotor  cortex, prefrontal cortex) |  |  |  |  | ╳  (insula) |  | ╳  (caudate nuclei and putamen, left caudate head) |  |  |  |  |  |
| Burton et al (2004) Brain | ╳  (right frontal lobe) | ╳  (bilateral temporal lobe) | ╳  (left parietal  lobe) |  | ╳  (occipital lobe) |  |  |  |  | ╳  (hippocampus and parahippocampal gyrus) |  |  |  |
| Price et al. (2004) NeuroImage |  |  |  |  |  |  |  | ╳  (midbrain) | ╳  (midbrain) |  |  | ╳  (cerebral peduncles) |  |
| Ramírez-Ruiz et al (2005) Journal of Neuroscience |  | ╳  (**without dementia**: temporal lobe) |  | ╳  (**without dementia**: limbic and paralimbic regions) | ╳  (**without dementia**: occipital lobe) |  |  |  |  |  |  |  |  |
| Chebrolu et al (2006) Experimental Neurology |  |  |  |  |  |  |  |  |  |  |  |  | ╳  Cerebellum (No difference) |
| Beyer et al. (2007) Neurology  (PDD is control group) |  |  |  |  |  |  |  |  |  |  |  |  |  |
| Bouchard et al. (2008) Neurobiology of Aging |  |  |  |  |  |  |  |  |  | ╳ | ╳ |  |  |
| Feldmann et al (2008) Movement Disorders | ╳  (bilateral orbitofrontal) | ╳  (right temporal regions) |  | ╳  (limbic system) |  |  |  |  |  |  |  |  |  |
| Ibarretxe-Bilbao et al. (2008) Journal of Neuroscience |  |  |  |  |  |  |  |  |  | ╳  (hippocampal Head) |  |  |  |
| Beninger et al. (2009) Journal of Neurology |  |  | ╳  (right quadrangular lobe) |  |  |  |  |  |  |  |  |  | ╳ |
| Cardoso et al. (2009) NeuroImage | ╳  (medial prefrontal cortex) |  |  |  |  |  |  | ╳  (putaminal grey matter loss) | ╳  (left mediodorsal (MD) thalamus ↓  **mediodorsal thalamic nuclei bilaterally ↑)** |  |  |  |  |
| Camicioli et al. (2009) Parkinsonism and Related Disorders |  | ╳  (MT, right temporal lobe) | ╳  (left Precuneous) |  | ╳  (left uncus, fusiform gyri) |  |  | ╳  (left putamen, caudate) |  |  |  |  | ╳ |
| Ibarretxe-Bilbao et al. (2009) European Journal of Neuroscience | ╳  (orbitofrontal cortex) |  |  |  |  |  |  |  |  |  | ╳  (amygdala) |  |  |
| Jubault et al. (2009)  PLoS ONE |  |  |  |  |  |  |  |  |  |  |  | ╳  (pons and the medulla oblongata) |  |
| Martin et al. (2009) Movement Disorder |  | ╳  (superior temporal lobe) |  |  | ╳  (anterior right fusiform gyri) |  |  |  |  |  |  |  |  |
| Wattendorf et al (2009) Journal of Neuroscience | ╳  (right piriform cortex) | ╳  (right piriform cortex) |  | ╳ (olfactory-eloquent regions of the limbic and paralimbic cortex) |  |  |  |  |  |  | ╳  (right  amygdala) |  |  |
| Agosta et al. (2010) European Journal of Neuroscience  (PSP-RS) | ╳  (orbitofrontal, prefrontal and precentral ⁄ premotor  regions) |  |  |  |  | ╳  Internal Capsulae |  | ╳  (midbrain) | ╳  (midbrain) |  |  |  | ╳  (left superior cerebellar peduncle) |
| Agosta et al. (2010) European Journal of Neuroscience  (PSP-P) | ╳  (bilateral frontal cortex) | ╳ |  |  |  | ╳  Internal Capsulae |  | ╳  (central midbrain, caudate nucleus) | ╳  (central midbrain, | ╳  (right hippocampus) |  |  | ╳  (left cerebellar lobe and dentate nuclei) |
| Borghammer et al. (2010) European Journal of Neurology | ╳ | ╳ |  |  |  |  |  |  |  |  |  |  | ╳  (left cerebellum, ) |
| Draganski & Bhatia (2010) Movement disorders Review Article |  |  |  | ╳  (limbic  cortex) |  |  |  | ╳  (basal ganglia) |  |  |  |  |  |
| Hamasaki et al. (2010) Acta Neurochir | ╳  **(White Matter:** ganglia-thalamocortical circuit)  Positively correlated with STN stimulation |  |  |  |  |  |  | ╳  **(White Matter:** ganglia-thalamocortical circuit)  Positively correlated with STN stimulation | ╳  **(White Matter:** ganglia-thalamocortical circuit)  Positively correlated with STN stimulation |  |  |  |  |
| Focke et al. (2011) Human Brain Mapping |  |  |  |  |  |  |  | ╳  putaminal  grey matter | ╳  (matter loss in the mesencephalon |  |  |  | ╳  (cerebellar grey matter) |
| Ibarretxe-Bilbao et al. (2011) Journal of Neurology, Neurosurgery, and Psychiatry | ╳  (limbic, paralimbic) and neocortical areas | ╳  (limbic, paralimbic) and  neocortical areas | ╳  (limbic, paralimbic) and  neocortical areas | ╳  (limbic, paralimbic) and  neocortical areas |  | ╳  (limbic, paralimbic) and  neocortical areas | ╳  (limbic, paralimbic) and  neocortical areas |  |  | ╳  (limbic, paralimbic) and  neocortical areas |  |  |  |
| Ibarretxe-Bilbao et al. (2011) Journal of the Neurological Sciences  Review Article | ╳  (frontal-striatal circuit) | ╳  (temporal lobe) |  |  |  |  |  | ╳  (frontal-striatal circuit) |  |  |  |  |  |
