# Supplementary material for: VBM Reveals Brain Volume Differences between Parkinson’s Disease and Essential Tremor Patients
Source: Front Hum Neurosci. 2013 Jun 14;7:247. doi: 10.3389/fnhum.2013.00247 (PMC3682128; doi:10.3389/fnhum.2013.00247)
Supplement: Supplementary file 2 [file 46476_Duann_DataSheet2.DOC]

**Supplementary Table 2a**

**Brain Volume of Healthy Controls larger than that of Parkinson’s Disease with Basic VBM and DARTEL VBM Methods**

| **Brain Region (Hemisphere)** | **Cluster Size (Voxel)** | **T** | **P*_Uncorr_*** | **MNI coordinate**  **For the voxel with**  **local maxima** | | |
| --- | --- | --- | --- | --- | --- | --- |
|  |  |  |  | X | Y | Z |
| **Healthy Controls > Parkinson’s Disease Patients (Basic VBM)**  Note: (P*_uncorrected_* = 0.005, K = 30) | | | | | | |
| Superior Temporal Gyrus (L) | **434** | **5.390** | **0.000** | -50 | 4 | -4 |
| Inferior Parietal Lobule (L) | **60** | **4.555** | **0.000** | -50 | -48 | 28 |
| Lentiform Nucleus (L) | **826** | **4.535** | **0.000** | 28 | -4 | 14 |
| Temporal Pole (L) | **101** | **4.476** | **0.000** | -30 | 12 | -50 |
| Middle Frontal Gyrus (R) | **53** | **4.409** | **0.000** | 36 | 42 | 4 |
| Inferior Temporal Gyrus (L) | **32** | **4.393** | **0.000** | -52 | -28 | -30 |
| Insula (R) | **60** | **4.374** | **0.000** | 42 | 22 | 0 |
| Insula (L) | **241** | **4.244** | **0.000** | -34 | -20 | 10 |
| Middle Occipital Gyrus (R) | **67** | **4.216** | **0.000** | 42 | -68 | -8 |
| Medial Frontal Gyrus (L) | **258** | **4.173** | **0.000** | -4 | 54 | -10 |
| Cerebellum (Vermis_8 (aal) (L)) | **39** | **3.958** | **0.000** | -2 | -68 | -40 |
| Fusiform Gyrus (R) | **31** | **3.927** | **0.000** | 44 | -38 | -28 |
| Fusiform Gyrus (L) | **42** | **3.794** | **0.001** | -48 | -58 | -20 |
| Fusiform Gyrus (R) | **34** | **3.756** | **0.001** | 54 | -22 | -32 |
| Parahippocampal Gyrus (L) | **37** | **3.645** | **0.001** | -28 | -22 | -18 |
| Fusiform Gyrus (R) | **30** | **3.616** | **0.001** | 32 | -14 | -36 |
| Middle Frontal Gyrus (L) | **33** | **3.524** | **0.001** | -28 | 30 | 28 |
| Inferior Parietal Lobule (R) | **47** | **3.491** | **0.001** | 58 | -40 | 22 |
| Medial Frontal Gyrus (L) | **47** | **3.447** | **0.001** | -10 | 18 | -20 |
| Lingual Gyrus (R) | **86** | **3.437** | **0.001** | 12 | -34 | 4 |
| Superior Temporal Gyrus (R) | **78** | **3.381** | **0.001** | 50 | 2 | -10 |
| Parahippocampal Gyrus (R) | **77** | **3.219** | **0.002** | 20 | -10 | -28 |
| Lingual Gyrus (R) | **38** | **3.147** | **0.002** | 4 | -68 | -4 |
| **Healthy Controls > Parkinson’s Disease Patients (DARTEL VBM)**  Note: (P*_uncorrected_* =0.000001, K = 30) | | | | | | |
| Caudate Body (R) | 347 | 11.97 | 0.000 | 11 | -7 | 27 |
| Cerebellum, Inferior Semi-Lunar Lobule (Cerebelum_Crus2) (L) | 1923 | 11.16 | 0.000 | -50 | -68 | -51 |
| Middle Cingullum (R) | 213 | 9.96 | 0.000 | 12 | -29 | 35 |
| Posterior Cingulate Cortex (L) | 127 | 9.67 | 0.000 | -17 | -48 | 27 |
| Superior Parietal Lobule (L) | 155 | 9.27 | 0.000 | -42 | -51 | 71 |
| Claustrum (L) | 159 | 9.23 | 0.000 | -29 | -18 | 14 |
| Caudate Body (L) | 388 | 9.22 | 0.000 | -8 | 9 | 9 |
| Cerebellum Anterior Lobe (Cerebellum 10)(R) | 90 | 8.83 | 0.000 | 29 | -30 | -38 |
| Middle Frontal Gyrus (L) | 77 | 8.70 | 0.000 | -23 | 11 | 41 |
| Superior Parietal Lobule (R) | 111 | 8.42 | 0.000 | 38 | -56 | 71 |
| Middle Cingulum (L) | 96 | 8.40 | 0.000 | -12 | -18 | 36 |
| Middle Frontal Gyrus (L) | 104 | 8.01 | 0.000 | -48 | 50 | 30 |
| Cerebellum, Inferior Semi-Lunar Lobule (Cerebellum Crus2) (R) | 74 | 7.94 | 0.000 | 50 | -68 | -51 |
| Precentral Gyrus (R) | 115 | 7.46 | 0.000 | 63 | 9 | 35 |
| Middle Frontal Gyrus (L) | 36 | 7.11 | 0.000 | -41 | 66 | 11 |

**Supplementary Table 2b**

**Brain Volume of Parkinson’s Disease larger than that of Healthy Controls with Basic VBM and DARTEL VBM Methods**

| **Brain Region (Hemisphere)** | **Cluster Size (Voxel)** | **T** | **P*_Uncorr_*** | **MNI coordinate**  **For the voxel with**  **local maxima** | | |
| --- | --- | --- | --- | --- | --- | --- |
|  |  |  |  | X | Y | Z |
| **Parkinson’s Disease Patients > Healthy Controls** **(Basic VBM)**  Note: (P*_uncorrected_* = 0.005, K = 30) | | | | | | |
| Thalamus, Ventral Posterior Lateral Nucleus (R) | 296 | 4.253 | 0.001 | 22 | -22 | 8 |
| **Parkinson’s Disease Patients > Healthy Controls (DARTEL VBM)**  Note: (P*_uncorrected_* =0.000001, K = 30) | | | | | | |
| Middle Cingullum (L) | 138 | 13.391 | 0.000 | -21 | -18 | 48 |
| Cerebellum Anterior Lobe (Cerebellum 9) (R) | 149 | 11.583 | 0.000 | 20 | -44 | -38 |
| Inferior Parietal Lobule (L) | 36 | 11.069 | 0.000 | -41 | -35 | 32 |
| SMA (R) | 67 | 10.951 | 0.000 | 18 | -26 | 50 |
| Inferior Temporal Gyrus (R) | 1317 | 10.902 | 0.000 | 32 | -20 | -47 |
| SMA (R) | 95 | 10.343 | 0.000 | 14 | -3 | 50 |
| Medial Globus Pallidus (L) | 174 | 10.229 | 0.000 | -12 | -3 | -2 |
| Middle Frontal Gyrus (L) | 66 | 9.405 | 0.000 | -29 | 17 | 35 |
| Precentral Gyrus (R) | 139 | 8.782 | 0.000 | 32 | -14 | 29 |
| Thalamus, Ventral Posterior Lateral Nucleus (R) | 198 | 8.625 | 0.000 | 24 | -29 | 6 |
| Inferior Parietal Lobule (R) | 30 | 8.378 | 0.000 | 45 | -38 | 32 |
| Middle Frontal Gyrus (L) | 338 | 8.273 | 0.000 | -32 | -12 | 30 |
| Superior Frontal Gyrus (R) | 73 | 8.169 | 0.000 | 18 | 50 | 51 |
| Thalamus, Pulvinar (L) | 37 | 7.956 | 0.000 | -24 | -26 | 12 |
| Insula (R) | 42 | 7.836 | 0.000 | 40 | 5 | 15 |
| Inferior Temporal Gyrus (L) | 306 | 7.812 | 0.000 | -72 | -36 | -21 |
| Precuneus (R) | 41 | 7.710 | 0.000 | 1 | -50 | 80 |
| Superior Frontal Gyrus (L) | 35 | 7.250 | 0.000 | -30 | 44 | 48 |
| Middle Frontal Gyrus (R) | 43 | 7.023 | 0.000 | 18 | 69 | 26 |
