# Supplementary material for: VBM Reveals Brain Volume Differences between Parkinson’s Disease and Essential Tremor Patients
Source: Front Hum Neurosci. 2013 Jun 14;7:247. doi: 10.3389/fnhum.2013.00247 (PMC3682128; doi:10.3389/fnhum.2013.00247)
Supplement: Supplementary file 3 [file 46476_Duann_DataSheet3.DOC]

**Supplementary Table 3a**

**Brain Volume of Healthy Controls larger than that of Essential Tremor Patients with Basic VBM and DARTEL VBM Methods**

| **Brain Region (Hemisphere)** | **Cluster Size (Voxel)** | **T** | **P*_Uncorr_*** | **MNI coordinate**  **For the voxel with**  **local maxima** | | |
| --- | --- | --- | --- | --- | --- | --- |
|  |  |  |  | X | Y | Z |
| **Healthy Controls > Essential Tremor Patients (Basic VBM)**  Note: (P*_uncorrected_* = 0.01, K = 30) | | | | | | |
| Caudate Body (R) | 464 | 4.895 | 0.000 | 16 | 12 | 22 |
| Middle Temporal Pole (L) | 237 | 4.225 | 0.000 | -42 | 20 | -32 |
| Caudate Body (L) | 470 | 4.184 | 0.000 | -18 | 12 | 20 |
| Superior Occipital Lobe (R) | 1304 | 3.830 | 0.000 | 36 | -92 | 34 |
| Middle Occipital Gyrus (R) | 58 | 3.768 | 0.001 | 28 | -86 | 8 |
| Insula (L) | 125 | 3.540 | 0.001 | -34 | 24 | 8 |
| Medial Frontal Gyrus (L) | 33 | 3.539 | 0.001 | -12 | 22 | -18 |
| Precuneus (L) | 696 | 3.457 | 0.001 | -14 | -92 | 44 |
| Inferior Temporal Lobe (R) | 133 | 3.351 | 0.002 | 62 | -54 | -26 |
| Superior Temporal Gyrus (L) | 148 | 3.261 | 0.002 | -62 | -54 | 10 |
| Cuneus (R) | 88 | 3.249 | 0.002 | 12 | -74 | 42 |
| Middle Cingulum (L) | 33 | 3.229 | 0.002 | -8 | -10 | 42 |
| Insula (R) | 50 | 3.197 | 0.002 | 36 | 24 | 6 |
| Rolandic Operculum (L) | 54 | 3.092 | 0.003 | -60 | 4 | 2 |
| Superior Parietal Lobule (L) | 43 | 3.029 | 0.003 | -40 | -60 | 62 |
| Middle Temporal Gyrus (R) | 229 | 3.011 | 0.003 | 60 | -62 | -4 |
| Inferior Temporal Gyrus (L) | 83 | 2.892 | 0.004 | -56 | -58 | -20 |
| Inferior Parietal Lobule (R) | 42 | 2.820 | 0.005 | 60 | -30 | 30 |
| **Healthy Controls > Essential Tremor Patients (DARTEL VBM)**  Note: (P*_uncorrected_* = 0.000001, K = 30) | | | | | | |
| Caudate (R) | 2599 | 16.130 | 0.000 | 9 | 11 | 21 |
| Insula (L) | 253 | 13.020 | 0.000 | -30 | 27 | 14 |
| Lateral Globus Pallidus (L) | 148 | 11.213 | 0.000 | -23 | -9 | -2 |
| Cerebellum, Inferior Semi-Lunar Lobule (R) | 1046 | 10.034 | 0.000 | 4 | -69 | -54 |
| Medial Globus Pallidus (R) | 238 | 9.573 | 0.000 | 21 | -12 | -2 |
| Posterior Cingulate Cortex (R) | 53 | 8.971 | 0.000 | 12 | -50 | 21 |
| Cerebellum, Posterior Lobe (L) | 359 | 8.921 | 0.000 | -53 | -71 | -41 |
| Superior Parietal Lobule (L) | 460 | 8.920 | 0.000 | -27 | -71 | 63 |
| Cerebellum, Posterior Lobe (L) | 163 | 8.862 | 0.000 | 6 | -84 | -48 |
| Precentral Gyrus (R) | 58 | 8.847 | 0.000 | 33 | -7 | 32 |
| Cerebellar Tonsil (R) | 37 | 8.467 | 0.000 | 20 | -50 | -42 |
| Superior Parietal Lobule (R) | 83 | 8.228 | 0.000 | 17 | -69 | 66 |
| Middle Temporal Pole (L) | 56 | 8.225 | 0.000 | -42 | 29 | -32 |
| Insula (R) | 84 | 8.133 | 0.000 | 33 | 11 | 20 |
| Superior Parietal Lobule (R) | 160 | 8.067 | 0.000 | 38 | -84 | 56 |
| Superior Frontal Gyrus (L) | 30 | 7.857 | 0.000 | -17 | 5 | 53 |
| Precuneus (L) | 49 | 7.590 | 0.000 | -18 | -51 | 32 |
| Orbitofrontal Cortex (R) | 33 | 7.076 | 0.000 | 15 | 56 | -29 |
| Superior Temporal Gyrus (L) | 40 | 7.069 | 0.000 | -66 | -66 | 17 |

**Supplementary Table 3b**

**Brain Volume of Essential Tremors Patients larger than that of Healthy Controls with Basic VBM and DARTEL VBM Methods**

| **Brain Region (Hemisphere)** | **Cluster Size (Voxel)** | **T** | **P*_Uncorr_*** | **MNI coordinate**  **For the voxel with**  **local maxima** | | |
| --- | --- | --- | --- | --- | --- | --- |
|  |  |  |  | X | Y | Z |
| **Essential Tremor Patients > Healthy Controls (Basic VBM)**  Note: (P*_uncorrected_* = 0.01, K = 30) | | | | | | |
| Inferior Frontal Gyrus (R) | 82 | 4.163 | 0.000 | 34 | 28 | 16 |
| Middle Temporal Gyrus (L) | 78 | 3.774 | 0.001 | -46 | -60 | -2 |
| Precentral Gyrus (L) | 35 | 3.553 | 0.001 | -54 | 0 | 10 |
| Inferior Temporal Gyrus (R) | 91 | 3.312 | 0.002 | 48 | -54 | -8 |
| **Essential Tremor Patients > Healthy Controls (DARTEL VBM)**  Note: (P*_uncorrected_* = 0.000001, K = 30) | | | | | | |
| Middle Temporal Gyrus (R) | 214 | 14.308 | 0.000 | 41 | -48 | 0 |
| Inferior Parietal Lobule (R) | 160 | 11.704 | 0.000 | 32 | -48 | 29 |
| Subcallosal Gyrus (R) | 466 | 11.613 | 0.000 | -11 | 3 | -17 |
| Medial Temporal Lobe (R) | 1484 | 10.582 | 0.000 | -12 | 2 | -45 |
| Cerebellum Posterior Lobe  (Cerebellum 8) (R) | 188 | 10.055 | 0.000 | 15 | -66 | -39 |
| Lateral Globus Pallidus (L) | 59 | 9.240 | 0.000 | -17 | -1 | 8 |
| Inferior Frontal Gyrus (L) | 67 | 9.089 | 0.000 | -44 | 20 | -9 |
| Superior Frontal Gyru (R) | 190 | 9.072 | 0.000 | 25 | 44 | 48 |
| Inferior Frontal Triangular (L) | 30 | 8.764 | 0.000 | -38 | 20 | 20 |
| Middle Temporal Gyrus (L) | 68 | 8.724 | 0.000 | -41 | -51 | -5 |
| Inferior Parietal Lobule (R) | 34 | 8.570 | 0.000 | 39 | -30 | 30 |
| Subthalamic Nucleus (L) | 43 | 8.290 | 0.000 | -9 | -17 | -6 |
| Cerebellum Posterior Lobe  (Cerebellum 8) (L) | 249 | 8.279 | 0.000 | -23 | -63 | -44 |
| Postcentral Gyrus (L) | 54 | 8.268 | 0.000 | -26 | -38 | 44 |
| Precentral Gyrus (R) | 36 | 7.881 | 0.000 | 21 | -23 | 48 |
| Postcentral Gyrus (L) | 30 | 7.630 | 0.000 | -39 | -20 | 27 |
| Middle Frontal Gyrus (L) | 31 | 7.625 | 0.000 | -29 | 45 | 50 |
| Parahippocampus (L) | 36 | 7.579 | 0.000 | -15 | -17 | -29 |
| Middle Frontal Gyrus (L) | 46 | 7.147 | 0.000 | -50 | 17 | 54 |
