# Supplementary material for: VBM Reveals Brain Volume Differences between Parkinson’s Disease and Essential Tremor Patients
Source: Front Hum Neurosci. 2013 Jun 14;7:247. doi: 10.3389/fnhum.2013.00247 (PMC3682128; doi:10.3389/fnhum.2013.00247)
Supplement: Supplementary file 4 [file 46476_Duann_DataSheet4.DOC]

**Supplementary Table 4a**

**Brain Volume of Parkinson’s Disease Patients larger than that of Essential Tremor Patients with Basic VBM and DARTEL VBM Methods**

| **Brain Region (Hemisphere)** | **Cluster Size (Voxel)** | **T** | **P*_Uncorr_*** | **MNI coordinate**  **For the voxel with**  **local maxima** | | |
| --- | --- | --- | --- | --- | --- | --- |
|  |  |  |  | X | Y | Z |
| **Parkinson’s Disease Patients > Essential Tremor Patients (Basic VBM)**  Note: (P*_uncorrected_* = 0.005, K = 30) | | | | | | |
| Superior Temporal Lobe (R) | 167 | 5.419 | 0.000 | 70 | -26 | 18 |
| Thalamus, Lateral Posterior Nucleus (R) | 105 | 5.255 | 0.000 | 22 | -20 | 22 |
| Inferior Frontal Operculum (R) | 33 | 4.671 | 0.000 | 38 | 12 | 18 |
| Superior Occipital Gyrus (L) | 1532 | 4.386 | 0.000 | -40 | -90 | 30 |
| Superior Temporal Gyrus (R) | 43 | 3.818 | 0.001 | 62 | 8 | 2 |
| Pons (L) | 47 | 3.410 | 0.002 | -8 | -18 | -34 |
| Middle Temporal Gyrus (R) | 36 | 3.354 | 0.002 | 68 | -28 | -4 |
| Caudate (R) | 36 | 3.240 | 0.002 | 24 | 30 | 8 |
| Cerebellum Posterior Lobe (Cerebellum Crus1) (L) | 116 | 3.196 | 0.003 | -52 | -70 | -22 |
| **Parkinson’s Disease Patients > Essential Tremor Patients (DARTEL VBM)**  Note: (P*_uncorrected_* = 0.000001, K = 30) | | | | | | |
| Cerebellum Anterior Lobe(R) | 357 | 17.916 | 0.000 | 21 | -47 | -38 |
| Middle Temporal Gyrus (R) | 115 | 12.280 | 0.000 | 42 | 0 | -26 |
| Inferior Temporal Gyrus (L) | 2366 | 12.132 | 0.000 | -68 | -42 | -23 |
| Inferior Temporal Gyrus (R) | 1815 | 11.057 | 0.000 | 33 | -20 | -48 |
| Insula (R) | 84 | 10.270 | 0.000 | 35 | 15 | 18 |
| Subthalamic Nucleus (R) | 174 | 10.183 | 0.000 | 15 | -15 | -8 |
| Middle Occipital Gyrus (R) | 60 | 10.004 | 0.000 | 29 | -77 | 8 |
| Middle Occipital Gyrus (L) | 33 | 9.365 | 0.000 | -32 | -72 | 6 |
| Cerebellum Anterior Lobe (L) | 41 | 8.799 | 0.000 | -20 | -45 | -38 |
| Precentral Gyrus (R) | 40 | 8.721 | 0.000 | 40 | -4 | 24 |
| Middle Frontal Gyrus (L) | 57 | 8.682 | 0.000 | -32 | 14 | 38 |
| Medial Globus Pallidus (L) | 60 | 8.323 | 0.000 | -20 | -12 | -2 |
| Angular Gyrus (R) | 36 | 8.078 | 0.000 | 38 | -80 | 60 |
| Insula (L) | 35 | 7.724 | 0.000 | -30 | 27 | 14 |
| Thalamus, Pulvinar (R) | 38 | 7.340 | 0.000 | 24 | -29 | 6 |

**Supplementary Table 4b**

**Brain Volume of Essential Tremor Patients larger than that of Parkinson’s Disease Patients with Basic VBM and DARTEL VBM Methods**

| **Brain Region (Hemisphere)** | **Cluster Size (Voxel)** | **T** | **P*_Uncorr_*** | **MNI coordinate**  **For the voxel with**  **local maxima** | | |
| --- | --- | --- | --- | --- | --- | --- |
|  |  |  |  | X | Y | Z |
| **Essential Tremor Patients > Parkinson’s Disease Patients (Basic VBM)**  Note: (P*_uncorrected_* = 0.005, K = 30) | | | | | | |
| Fusiform Gyrus (R) | 96 | 6.507 | 0.000 | 34 | -12 | -42 |
| Angular Gyrus (R) | 75 | 6.313 | 0.000 | 48 | -48 | 24 |
| Superior Temporal Pole (L) | 154 | 5.123 | 0.000 | -28 | 10 | -24 |
| Postcentral Gyrus (L) | 103 | 5.019 | 0.000 | -54 | -8 | 14 |
| Superior Temporal Gyrus (L) | 142 | 5.012 | 0.000 | -52 | -50 | 20 |
| Middle Frontal Gyrus (L) | 125 | 4.935 | 0.000 | -28 | 6 | 46 |
| Middle Temporal Gyrus (L) | 121 | 4.684 | 0.000 | -44 | -58 | -2 |
| Caudate (R) | 70 | 4.658 | 0.000 | 18 | 8 | 10 |
| Inferior Temporal Gyrus (L) | 74 | 4.278 | 0.000 | -56 | -34 | -20 |
| Inferior Temporal Gyrus (R) | 40 | 4.277 | 0.000 | 54 | -18 | -24 |
| Inferior Frontal Gyrus (R) | 130 | 4.166 | 0.000 | 38 | 38 | 4 |
| Calcarine Fissure (L) | 96 | 4.070 | 0.000 | -14 | -82 | 6 |
| Middle Temporal Gyrus (R) | 40 | 3.936 | 0.000 | 46 | -6 | -18 |
| Middle Temporal Gyrus (R) | 37 | 3.795 | 0.001 | 46 | -50 | 0 |
| Cerebellum Posterior Lobe (Cerebellum Crus1) (L) | 171 | 3.618 | 0.001 | -24 | -80 | -28 |
| Insula (L) | 70 | 3.563 | 0.001 | -30 | -26 | 14 |
| Cerebellum Posterior Lobe (Cerebellum Crus1) (L) | 73 | 3.502 | 0.001 | -40 | -50 | -36 |
| Parahippocampus (R) | 34 | 3.449 | 0.001 | 16 | -38 | -10 |
| Mamillary Body | 39 | 3.145 | 0.003 | 2 | -6 | -14 |
| **Essential Tremor Patients > Parkinson’s Disease Patients (DARTEL VBM)**  Note: (P*_uncorrected_* = 0.000001, K = 30) | | | | | | |
| Middle Frontal Gyrus (L) | 1629 | 13.310 | 0.000 | -53 | 17 | 54 |
| Middle Cingulum (R) | 171 | 11.657 | 0.000 | 14 | -32 | 35 |
| Middle Temporal Pole (R) | 880 | 11.042 | 0.000 | 60 | 9 | -38 |
| Middle Temporal Gyrus (R) | 113 | 10.683 | 0.000 | 42 | -50 | 0 |
| Middle Frontal Gyrus (L) | 104 | 10.465 | 0.000 | -27 | 9 | 42 |
| Middle Frontal Gyrus (R) | 1010 | 10.174 | 0.000 | 58 | 18 | 47 |
| Insula (L) | 158 | 9.693 | 0.000 | -30 | -6 | 17 |
| Supramarginal Gyrus (R) | 49 | 9.031 | 0.000 | 29 | -53 | 32 |
| Middle Frontal Gyrus (R) | 61 | 8.966 | 0.000 | 26 | -12 | 41 |
| Middle Temporal Pole (R) | 256 | 8.658 | 0.000 | 11 | 15 | -38 |
| Cerebellum Posterior Lobe, (Cerebellum 8) (R) | 41 | 8.351 | 0.000 | 27 | -29 | -50 |
| Insula (R) | 37 | 8.338 | 0.000 | 35 | 35 | 9 |
| Middle Temporal Gyrus (L) | 41 | 8.284 | 0.000 | -42 | -48 | 12 |
| Middle Temporal Gyrus (L) | 31 | 8.208 | 0.000 | -41 | -54 | 0 |
| Middle Temporal Pole (L) | 140 | 8.191 | 0.000 | -8 | 3 | -39 |
| Cerebellum Posterior Lobe (Cerebellum 8) (R) | 75 | 8.002 | 0.000 | 23 | -63 | -45 |
| Inferior Frontal Gyrus (L) | 39 | 7.992 | 0.000 | -60 | 26 | -5 |
| Cerebellum Posterior Lobe (Cerebellum 8) (L) | 45 | 7.929 | 0.000 | -20 | -66 | -44 |
